# New Antipoverty Drugs, Vaccines, and Diagnostics: A Research Agenda for the US President's Global Health Initiative (GHI)

**DOI:** 10.1371/journal.pntd.0001133

**Published:** 2011-05-31

**Authors:** Peter J. Hotez

**Affiliations:** 1 Department of Microbiology, Immunology, and Tropical Medicine, George Washington University, Washington, D.C., United States of America; 2 Sabin Vaccine Institute, Washington, D.C., United States of America

*Allocating just 1%–2% of Global Health Initiative funds to conduct research and development for neglected tropical diseases drugs, vaccines, and diagnostics would create a new generation of tools to eliminate our planet's greatest scourges and help shape United States foreign policy.*

On May 5, 2009, the Obama Administration announced its intention to launch an ambitious United States governmental strategy for global health [1]–[3]. The US Global Health Initiative (GHI) proposes US$63 billion over 6 years (FY 2009–FY 2014), US$10.5 billion annually on average, approximately 70% of which would be spent on the US President's Emergency Plan for AIDS Relief (PEPFAR) [1]. If appropriated each year by Congress, the GHI would represent a significant response to calls by the Institute of Medicine of the National Academies for the US government (USG) to invest US$15 billion annually on development assistance for global health by 2012 [3].

In its current form, most of GHI is devoted to direct implementation of existing treatments and preventive interventions for the “big three diseases,” i.e., HIV/AIDS, malaria, and tuberculosis, especially the delivery of antiretroviral drugs and other prevention measures, antimalarial drugs and bednets, and direct observed therapy, respectively, as well as other critical interventions to improve maternal and child health and strengthen health systems [1], [3]. There is also an unprecedented commitment to provide treatments for the neglected tropical diseases (NTDs), with US$65 million committed in FY2010 for rapid impact packages and related measures targeting the seven most common NTDs, which comprise the most prevalent infections affecting the world's poor [4]–[8].

## The US Commitment to Neglected Diseases R&D

GHI is already making a huge difference in the lives of the world's 1.4 billion poorest people in developing countries who live below the World Bank poverty figure of US$1.25 per day—a group sometimes referred to as the “bottom billion” [4]. However, currently GHI largely fails to address research and development (R&D) needs for the manufacture and testing of a new generation of global health products, i.e., new drugs, vaccines, diagnostics, and other tools, for neglected diseases, defined broadly here to include both the big three diseases and the NTDs [9]. To be sure, outside of GHI, the USG's overall investment in neglected diseases runs deep [9]. According to the 2009 G-FINDER (Global Funding of Innovation for Neglected Diseases) report in 2008, the USG provided almost three-quarters of all global public spending on neglected diseases, with an estimated approximate investment of US$1.25 billion [3], [9]. Approximately 86% (US$1.08 billion) of those funds came from the National Institutes of Health (NIH) and most of that from the National Institute of Allergy and Infectious Diseases (NIAID), with 80% of the NIAID funds committed for the big three diseases [9]. NIH-NIAID currently provides intramural support for the Dale and Betty Bumpers Vaccine Research Center (VRC), whose primary mission is to develop global HIV/AIDS vaccines [10], and the Laboratory of Malaria Immunology and Vaccinology [11], as well as substantial extramural support to universities and private research institutes to support basic research, the development of new drugs to overcome resistance [12]–[15], and some vaccine research. In addition, the United States Agency for International Development (USAID) provides substantial resources to support vaccine development for HIV/AIDS through the International AIDS Vaccine Initiative, a non-profit product development partnership (PDP) [16], and for malaria vaccine development in collaboration with the Walter Reed Army Institute for Research [17].

In 2008, over 60 ministers of health, science, technology, and education met in Bamako, Mali, for a Global Ministerial Forum on Research for Health [18]. The resulting call to action asked countries to commit themselves to allocate at least 2% of national health budgets to research, while funders such as the USG were asked to invest at least 5% of health sector aid for research [18]. The research funds provided by NIH alone (and indeed just NIAID commitment) are sufficient to meet the challenge laid out in Mali, and altogether the USG has spent US$1.25 billion annually on neglected disease research, the equivalent of approximately 12% of funds spent annually on GHI.

## R&D Targeted Specifically for the NTDs

A closer analysis of the USG's commitment to global health research reveals that only a very small percentage of funds for R&D were spent on the NTDs, with minimal support for the PDPs that produce new products for these conditions. Thus, while the USG invests heavily for neglected diseases R&D, there is a specific gap for NTD product support. Why is this significant?

The NTDs are primarily parasitic and bacterial infections, which together with dengue fever represent the most common conditions of the bottom billion [4]–[6]. The seven most common NTDs include the three major soil-transmitted helminthiases—ascariasis, hookworm infection, and trichuriasis (600–800 million cases for each helminth infection worldwide)—followed by schistosomiasis (200–600 million infections), lymphatic filariasis (120 million), trachoma (40 million), and onchocerciasis (20–40 million), followed by liver fluke infection (20 million), leishmaniasis (12 million), and Chagas disease (8–9 million) [19]–[22]. However, an unknown number of people, possibly as many as 50 million, may also be infected with amebiasis and dengue fever [23], [24]. Practically speaking, these huge numbers mean that virtually all of the bottom billion, i.e., all of the world's poor, suffers from at least one NTD. Moreover, the disabling effects of NTDs in children, pregnant women, and agricultural workers have been shown to produce a profound economic impact that actually traps the bottom billion in poverty [7], [19].

While the USG (through the NIH) and the major pharmaceutical companies are engaged in a global enterprise for developing new drugs and vaccines for HIV/AIDS, malaria, and tuberculosis, by comparison NTD product development is being neglected [6]. A list of the most urgently needed new control tools for the NTDs was highlighted previously [6]. The priority list includes safer and more effective drugs for kinetoplastid infections, such as Chagas disease, leishmaniasis, and human African trypanosomiasis; a macrofilaricide drug; and new vaccines to combat leishmaniasis, Chagas disease, hookworm infection, schistosomiasis, dengue, and enteric bacterial pathogens [6]. Such biotechnologies have been referred to as “antipoverty vaccines,” a term that reflects the reality that most of the NTDs actually cause poverty because of their adverse impact on child development and cognition and worker productivity. Thus, NTD vaccines (and presumably drugs and diagnostics as well) represent critical interventions for promoting economic development as well as health in low-income countries [4], [7], [8]. A more complete list of needed antipoverty technologies is provided in Table 1 [6], [9].

**Table 1:** New products required or under development for the major NTDs.^a^

Disease	New Drugs	New Vaccines	New Diagnostics	New Vector Control Products or Zoonotic Animal Reservoir Products to Block Transmission to Humans
**Protozoan NTDs**				
Amebiasis	−	+	+	−
Balantidiasis	−	−	−	−
Chagas disease	+	+	+	+
Giardiasis	−	−	+	−
Hum. African trypanosomiasis	+	+	+	+
Leishmaniasis	+	+	+	+
**Heliminth NTDs**				
Taeniasis-cysticercosis	+	−	+	+
Dracunculiasis	−	−	−	−
Echinococcosis	+	−	+	+
Food-borne trematodiases	+	+	+	+
Loiasis	+	−	+	−
Lymphatic filariasis	+	−	+	+
Onchocerciasis	+	+	+	+
Schistosomiasis	+	+	+	+
Ascariasis	−	−	+	+
Hookworm	+	+	+	−
Trichuriasis	+	−	+	−
Strongyloidiasis	+	+	+	−
Toxocariasis	+	−	+	+
**Viral NTDs**				
Dengue and other flaviviruses	+	+	+	+
Rabies	+	+	+	+
Rift Valley fever	+	+	+	+
**Bacterial NTDs**				
Baronellosis	+	−	+	−
Bovine tuberculosis	+	+	+	+
Buruli ulcer	+	+	+	−
Cholera	+	+	+	−
Enteric pathogens (Gram neg)	+	+	+	−
Leprosy	+	+	+	−
Leptospirosis	+	+	+	−
Rheumatic fever	−	+	−	−
Trachoma	−	+	+	−
Treponematoses	+	+	+	−
**Fungal NTDs**				
Mycetoma	+	−	+	−
Paracoccidiomycosis	+	+	−	−
**Ectoparasitic infections**	+	−	+	−

a List of NTDs modified from http://www.plosntds.org/static/scope.action.

+, New product needed or under development; −, new product not required or need not yet determined, based on information compiled from [6], [9], and the additional opinions of the author.

Because the NTDs occur almost exclusively among the bottom billion, most of the antipoverty technologies have no commercial value even though they offer the promise of tremendous public health benefit [6]–[9]. It follows that in the absence of substantial financial returns, with a few exceptions (such as the development of a vaccine for dengue, which also has a potential market for Singapore, the Gulf Coast of the United States, and the wealthier Brazilian coastal cities, for example), most of the major pharmaceutical companies have not embarked on substantial R&D programs for NTD products. Instead, today many of the antipoverty technologies are being developed by PDPs, i.e., non-profit organizations that employ industrial business practices in order to develop new technologies for neglected diseases [25], together with scientific R&D institutes and organizations in disease-endemic countries. Today, the PDPs depend on support from European governments in addition to substantial funds from the Bill & Melinda Gates Foundation [25], with comparatively modest support from the USG.

Thus, while the NIH is a significant contributor to global health research, the agency spends a high percentage of its funds on the big three diseases, with less than 10% of its overall neglected disease research budget to fund the most common NTDs, including the kinetoplastid infections ($49 million), dengue ($27 million), and all of the helminth infections ($23 million). Moreover, most of these NIH funds are allocated to basic research and not product development. In addition, USAID provides no funds for PDPs committed to the NTDs. This situation has started to turn around with a new effort by NIAID to fund PDPs [26], together with two decades of support for overseas Tropical Medicine Research Centers [27], but overall the USG, and USAID in particular, has not made major commitments to PDPs for NTD product development and clinical trials. In contrast, several European governments, including the British Department for International Development [28] and the Dutch Ministry of Foreign Affairs [29], have recently committed substantial PDP support, as well as the Brazilian Ministry of Health, which now supports PDPs for NTDs [30].

Overall, it has been estimated that approximately US$1 billion per year over the next 10 years will be required to put experimental treatments and vaccines in the PDP pipeline through large human trials and file them with regulators [31]. Other unpublished estimates have quoted considerably higher dollar amounts. Ultimately, a significant portion of this level of support could be provided by the USG, as well as European governments and the European Commission, and even some emerging market economies [32]. The Institute of Medicine of the National Academies 2009 report, *The U.S. Commitment to Global Health: Recommendations for the Public and Private Sectors*, specifically recommended support for PDPs committed to developing novel global health technologies and interventions [3].

## R&D for Vaccine Diplomacy

There are several important reasons why the USG should support NTD product development and testing by providing funds for both PDPs and for science and technology agencies of NTD-endemic countries. These activities are consistent with our nation's humanitarian principles because there is a key human rights dimension to NTD mitigation [33]. It has been previously argued that just as the world's poorest people have a fundamental right to have access to essential medicines, they also have rights to biomedical innovation [6]. But even beyond this humanitarian rationale there is an equally important element of enlightened self-interest for the USG and other governments to invest in R&D for antipoverty technologies.

The control and elimination of the NTDs potentially has US foreign policy implications. Most of the world's NTDs are believed to occur in areas of greatest US geopolitical interests [5]. The most heavily affected nations include those comprising the Organisation of the Islamic Conference, as some of the worst affected nations include the poorest Islamic countries, such as Indonesia, Bangladesh, Sudan, Mali, and Chad [34]; they also include powerful middle-income nations with nuclear weapons capabilities such as India, Pakistan, Iran, and North Korea [35]. Additionally, a further relationship has been noted between nations with NTDs and conflict such that the countries with the highest prevalence of NTDs are the most likely to have been engaged in war over the last two decades [36]. Indeed, the links between geopolitical interests, conflict, and neglected diseases provide a rationale for launching the GHI under the auspices of USAID and the Department of State, rather than through the NIH, CDC, or other agencies of the Department of Health and Human Services.

Because the NTDs have such a major geopolitical dimension, R&D for new antipoverty vaccines and drugs may therefore represent more than simply promoting new technologies for improving health. Instead, over the next decade the antipoverty technologies could emerge as powerful new interventions to enhance US foreign policy. I have used the term “vaccine diplomacy” to describe joint R&D activities between nations, especially those with major ideological differences [37]–[40]. This concept arises in part from an interesting Cold War history that led to the joint US–Soviet development of the oral polio vaccine [37]–[40]. With this paradigm in mind, could GHI funds be spent in order for American scientists to conduct similar science and technology diplomacy with selected middle-income countries, including some so-called innovative developing countries, specifically those with both high rates of NTDs and a sophisticated infrastructure for conducting scientific R&D [39]–[41]?

## Concluding Remarks

Setting aside approximately 1%–2% of the GHI (roughly US $100–200 million annually) for R&D on new antipoverty vaccines and drugs would dramatically increase the current support for new NTD antipoverty technologies, and simultaneously provide capacity building activities for key disease-endemic countries of strategic interest to the US. It could also provide a new and exciting role for PDPs committed to the NTDs, many of which are US based, to engage in vaccine diplomacy, and ultimately lead to the development of a new generation of poverty-reducing biotechnologies. The mechanisms by which funds are distributed could require the establishment of peer-reviewed study sections, possibly not too dissimilar to those established by the NIH in order to ensure that only the best science is funded, and in addition there could be specific requirements and oversight to place the science in a diplomatic context. There are also opportunities to bring in key international agencies and organizations, including WHO-TDR, the Special Programme for Research and Training in Tropical Diseases [42], and IVI, the International Vaccine Institute based in Korea and supported in part by the United Nations Development Program [43]. Such science and technology diplomatic outreach could lead to new peacetime roles for foreign scientists currently engaged in nuclear weapons development, meet President Obama's 2009 challenge in Cairo when he called on the US to reach out to the Islamic world [44], and simultaneously create a new dimension in US foreign policy that also plays to America's great strengths and intellectual prowess in biomedical R&D.
